# Temporal and Spatial Variation in the Abundance of Total and Pathogenic *Vibrio parahaemolyticus* in Shellfish in China

**DOI:** 10.1371/journal.pone.0130302

**Published:** 2015-06-10

**Authors:** Haihong Han, Fengqin Li, Weixing Yan, Yunchang Guo, Ning Li, Xiumei Liu, Jianghui Zhu, Jin Xu, Yan Chen, Xiugui Li, Hong Lv, Yiqian Zhang, Te Cai, Yuzhen Chen

**Affiliations:** 1 National Institute for Nutrition and Health, Chinese Center for Disease Control and Prevention, Beijing, China; 2 Key Laboratory of Food Safety Risk Assessment of Ministry of Health, China National Center for Food Safety Risk Assessment, Beijing, China; 3 Institute of Microbiological Examination, Guangxi Regional Center for Disease Control and Prevention, Nanning, China; 4 Institute of Microbiological Examination, Sichuan Provincial Center for Disease Control and Prevention, Chengdu, China; 5 Faculty of Sciences, University of Waterloo, Waterloo, Canada; 6 Department of Testing, Dalian Xigang District Center for Disease Control and Prevention, Dalian, China; 7 Institute of Bacterial Infectious Disease Control, Shandong Provincial Center for Disease Control and Prevention, Jinan, China; Fish Vet Group, THAILAND

## Abstract

We investigated the abundance of total and pathogenic *Vibrio parahaemolyticus* in shellfish sampled from four provinces in China during May 2013 and March 2014 using the most probable number-polymerase chain reaction (MPN-PCR) method. Total *V*. *parahaemolyticus* was detected in 67.7% of 496 samples. A total of 38.1% and 10.1% of samples exceeded 1,000 MPN g^-1^ and 10,000 MPN g^-1^, respectively. *V*. *parahaemolyticus* densities followed a seasonal and geographical trend, with Guangxi and Sichuan shellfish possessing total *V*. *parahaemolyticus* levels that were 100-fold higher than those of the Liaoning and Shandong regions. Moreover, the levels of *V*. *parahaemolyticus* were at least 10-fold higher in the summer and autumn than in the cooler seasons. Pathogenic *V*. *parahaemolyticus* levels were generally lower than total *V*. *parahaemolyticus* levels by several log units and tended to be high in samples contaminated with high total *V*. *parahaemolyticus* levels. The aqua farms had a lower prevalence but higher abundance of total *V*. *parahaemolyticus* compared to retail markets. The catering markets showed the lowest levels of total *V*. *parahaemolyticus*, but 20.0% of samples exceeded 1,000 MPN g^-1^. The levels of both total and pathogenic *V*. *parahaemolyticus* in oysters were higher than in clams. The log-transformed abundance of *V*. *parahaemolyticus* was significantly correlated with both water temperature and air temperature but not water salinity. These results provide baseline contamination data of *V*. *parahaemolyticus* in shellfish in China, which can be applied to local risk assessments to prioritize risk control to key sectors and evaluate the effectiveness of future control measures.

## Introduction

*Vibrio parahaemolyticus* is a halophilic, Gram-negative foodborne pathogen that has raised global concerns since the occurrence of the O3:K6 strain pandemic in 1996 [1]. It has since caused outbreaks in many parts of the world, including Europe, North America, and Southeast Asia [2–6]. *V*. *parahaemolyticus* is naturally present in areas used to grow and harvest shellfish, and it is considered to be part of the autochthonous microflora in estuarine and coastal environments [7]. *V*. *parahaemolyticus* is often present during warmer months in temperate waters. Its prevalence and abundance are influenced by two major factors: temperature and salinity [8].

Generally, human infections with *V*. *parahaemolyticus* are caused by the consumption of raw or undercooked seafood [9]. Molluscan shellfish, such as oysters, accumulate microorganisms from the environment during filter feeding, which is of concern because these shellfish are commonly ingested raw [10]. Moreover, shellfish undergo a complex farm-to-fork process that includes harvesting, storage, transportation, and consumption, and the growth and proliferation of *V*. *parahaemolyticus* can be either enhanced or suppressed at each stage. *V*. *parahaemolyticus* can multiply rapidly in shellfish post-harvest and reach a sufficient level to cause food poisoning if the shellfish are not instantly refrigerated [11].

Although many infections have been epidemiologically linked to the consumption of seafood contaminated with pathogenic strains, virulent strains of *V*. *parahaemolyticus* are rarely isolated from food [12]. The selective detection and enumeration of virulent strains in food and environmental samples are difficult due to the relatively low population densities of virulent strains and the similar growth kinetics between virulent and avirulent strains [13].

The efficient detection of *V*. *parahaemolyticus* requires the recognition of several targets. The nonpathogenic product of the thermolabile hemolysin gene (*tlh*) is a useful target for the detection of total *V*. *parahaemolyticus* [14, 15]. In contrast, thermostable direct hemolysin (TDH) and TDH-related hemolysin (TRH) are two major virulence factors associated with *V*. *parahaemolyticus*-mediated disease [16]. The *V*. *parahaemolyticus* pandemic group strains include O3:K6 and its derivatives (the O4:K68, O1:K25, and O1:KUT serotypes), which have emerged since 1996 [17]. Their common genetic marker ORF8 is a unique open reading frame of the filamentous phage f237 [18, 19]. The majority of clinical strains carry either one or both of the *tdh* and *trh* genes, whereas the presence of these genes is much lower in environmental isolates [20, 21]. Therefore, the presence of one or more of the *tdh*, *trh*, and ORF8 genes is routinely used for indicating whether a strain is pathogenic [22].

The real-time polymerase chain reaction (PCR) method developed by Ward et al. [23], which targets the four genes mentioned above, is capable of specifically and sensitively detecting total and pathogenic *V*. *parahaemolyticus* in a rapid and reliable manner. Herein, the most probable number (MPN) method was combined with the real-time PCR protocol to perform a quantitative analysis of total and pathogenic *V*. *parahaemolyticus* associated with molluscan shellfish. Four provinces in China with shellfish industries were surveyed: Liaoning, which is located in the south of northeastern China, bordered by the Yellow and Bohai Seas in the south and facing the Shandong Peninsular across Bohai Bay; Shandong, a major coastal province in eastern China that borders the Bohai and Yellow Seas in the east; Sichuan, an inland province in southwestern China; and The Guangxi Zhuang Autonomous Region, located in the west of southern China along the coast of the Beibu gulf. This paper describes the levels of *V*. *parahaemolyticus* detected in oysters and clams sampled over four seasons at aqua farms, retail markets, and catering markets in these four provinces in China to determine the temporal and spatial distribution of *V*. *parahaemolyticus* and assess the public health risk associated with shellfish consumption in these regions.

## Material and Methods

### Sampling

A total of 496 oyster and clam samples were collected from three market categories (aqua farms, retail markets, and catering markets) in four provinces (Liaoning, Guangxi, Sichuan, and Shandong) between May 2013 and March 2014. Each month, 10 samples from one of two aqua farms, 10–15 samples from retail markets, and three samples from catering markets were collected in at least one of the four provinces of interest. The number of samples in each category is specified in Table 1. Aqua-farm shellfish consisted exclusively of oysters from the Liaoning and Guangxi provinces collected immediately after harvest. Samples from retail and catering markets included clams and oysters obtained from all four provinces; the shellfish from catering markets were either opened onsite or half-baked. Due to the inland location of Sichuan, only iced shellfish were available in the retail markets, with most shellfish transported from Guangxi.

**Table 1:** Abundance of *V*. *parahaemolyticus* in shellfish.

Characteristics (No. of samples)	% of samples with total and pathogenic *V*. *parahaemolyticus* densities within the indicated MPN g^-1^ ranges						Mean densities of *tlh* positive samples	Mean densities of all samples
	None detected	<10	>10 to 10^2^	>10^2^ to 10^3^	>10^3^ to 10^4^	>10^4^ to 10^5^	Mean±SD ^a^	Mean±SD ^a^
Pathogenic (496)	77.4	10.9	5.6	2.6	3.4	0.0	21.2±13.8	0.5±11.2
Total (496)	32.3	8.5	8.3	12.9	28.0	10.1	660.7±13.5	43.7±87.1
Sample sites								
Liaoning (132)	67.4	11.4	3.8	0.8	16.7	0.0	151.4±16.2	1.4±37.2
Guangxi (183)	15.8	0.0	4.4	22.4	32.2	25.1	2818.4±5.0	602.6±49.0
Sichuan (131)	12.2	11.5	18.3	13.7	41.2	3.1	302.0±11.5	120.2±29.5
Shandong (50)	52.0	24.0	8.0	8.0	8.0	0.0	30.9±12.6	1.9±24.5
Sample species								
Clams (129)	45.0	14.7	15.5	5.4	19.4	0.0	97.7±12.6	5.4±41.7
Oysters (367)	27.8	6.3	5.7	15.5	31.1	13.6	1096.5±10.7	93.3±87.1
Market categories								
Retail market (226)	23.5	11.9	13.3	9.7	28.3	13.3	467.7±17.4	70.8±69.2
Aqua farm (180)	31.7	4.4	1.7	19.4	32.2	10.6	1479.1±7.1	81.3±97.7
Catering market (90)	55.6	7.8	8.9	7.8	18.9	1.1	218.8±13.2	3.8±55.0
Seasons								
Summer (202)	31.2	3.5	4.0	15.3	38.6	7.4	1174.9±7.4	72.4±87.1
Autumn (136)	19.1	5.9	8.8	15.4	33.8	16.9	1174.9±11.0	213.8±63.1
Winter (70)	38.6	30.0	7.1	7.1	11.4	5.7	66.1±19.5	6.3±43.7
Spring (88)	50.0	6.8	18.2	8.0	8.0	9.1	229.1±18.2	5.9±67.6

^a^ SD: standard deviation.

Samples were collected separately in sterile bags and placed on ice immediately. Water salinity and temperature were measured with a digital salinity meter (ATAGO, PAL-06S). The air temperature was also recorded for a portion of samples.

### Sample Preparation and MPN-Multiplexed Real-Time PCR Assay

The samples were washed in running potable water and shucked using aseptic techniques within 4 h of collection. A total of 25 g of meat and intervalve liquid homogenate from the shellfish was blended with 225 mL of alkaline peptone water (APW) to generate a 1:10 dilution. Three serial 10-fold dilutions (10^–1^ to 10^–5^) were used following the MPN culture method (three tubes, three dilutions). A total of 1.5 mL of the MPN culture was collected by centrifugation, and the pellet was then resuspended and centrifuged twice. Finally, the pellet was resuspended in 200 μL of TE buffer and heated to 100°C for 10 min to facilitate cell lysis and DNA release.

Four primer pairs and probes for detection of the *tlh*, *tdh*, *trh* and ORF8 genes were synthesized, and the reactions were performed as described by Ward et al. [23]. Lysis liquid spiked with purified genomic DNA targeting the four genes was amplified as an internal control to eliminate false-negative results due to inhibition by the shellfish tissue matrix.

### Statistical analysis

The MPN value was normalized as follows: An MPN value < 0.3 MPN g^-1^ was estimated to be 0.15 MPN g^-1^, while MPN values > 110, > 1,100, and > 11,000 MPN g^-1^ were assigned as 240, 2,400, and 24,000 MPN g^-1^, respectively (and assumed to equal MPN values of 3, 3, and 0, respectively, for the next dilution). A positive result for pathogenic *V*. *parahaemolyticus* was defined by the presence of one or more of the *tdh*, *trh* and ORF8 targets, whereas a negative result was defined by the presence of only the *tlh* gene. For the seasonal analysis, Spring was defined as March to May, Summer as June to August, Autumn as September to November, and Winter as December to February. The densities of *V*. *parahaemolyticus* in the shellfish samples were log_10_ transformed to normalize the data for analysis.

The prevalence of *V*. *parahaemolyticus* in the shellfish samples was analyzed by Pearson’s chi-square test. The Pearson’s correlation method was applied to examine the association between the total *V*. *parahaemolyticus* abundance and pathogenic *V*. *parahaemolyticus* levels. Difference in *V*. *parahaemolyticus* abundance among regional/seasonal categories were tested using analysis of variance (ANOVA). Multivariable logistic regression analysis was used to examine the association between either the total *V*. *parahaemolyticus* densities or the pathogenic *V*. *parahaemolyticus* densities with possible explanatory variables, including sampling site, sample species, market category and sampling season. A backwards-elimination approach was applied during the model-building process that initially included all variables in the model; the significance of each variable was subsequently assessed using the likelihood-ratio test statistic (LRS). Total *V*. *parahaemolyticus* densities were analyzed by regression of the mean log_10_ densities of the samples against temperature and salinity. A *P*-value of 0.05 was chosen as the significance level.

## Results

Among the 496 samples, 67.7% (336 out of 496) were identified as *V*. *parahaemolyticus-*positive (recognized as *tlh-*positive), 15.5% (77 out of 496) were identified as *tdh*-positive, 10.7% (53 out of 496) were identified as *trh*-positive, and 10.9% (54 out of 496) were identified as ORF8-positive. Furthermore, 22.6% (112 out of 496) of the samples contained pathogenic *V*. *parahaemolyticus*, and 11.3% (56 out of 496) bore two or three toxic genes, representing 50% (56 out of 112) of the pathogenic isolates.

Table 1 shows the percentages of samples with *V*. *parahaemolyticus* densities within the indicated MPN g^-1^ ranges. The density of total *V*. *parahaemolyticus* varied from < 0.3 to 46,000 MPN g^-1^. A total of 38.1% and 10.1% of the samples exceeded 1,000 MPN g^-1^ and 10,000 MPN g^-1^, respectively; all samples that exceeded 10,000 MPN g^-1^ were collected from either the Guangxi or Sichuan provinces. The mean level of the 336 *V*. *parahaemolyticus tlh-*positive samples was 660.7 MPN g^-1^, with a standard deviation of 13.5 MPN g^-1^; whereas the mean level of all 496 samples was 1 log lower (43.7 MPN g^-1^), with a larger standard deviation of 87.1 MPN g^-1^.

A significant regional trend was observed in the abundance of total *V*. *parahaemolyticus*, with shellfish from Guangxi (602.6 MPN g^-1^) and Sichuan (120.2 MPN g^-1^) having mean levels 100-fold higher than those from the Liaoning and Shandong regions (Table 1). Table 2 compares the mean levels of *V*. *parahaemolyticus* in shellfish by region and season. Total *V*. *parahaemolyticus* levels in the Guangxi and Sichuan regions were significantly higher than those in the Liaoning and Shandong regions in all seasons (Table 2). The geometric means of the total *V*. *parahaemolyticus* levels in the Guangxi and Sichuan regions were generally higher than 23 MPN g^-1^, even in the winter and spring, with the highest level of 1700 MPN g^-1^ detected in the Guangxi region during the summer (Table 2). Total *V*. *parahaemolyticus* mean densities in the Liaoning and Shandong regions were less than 4 MPN g^-1^ in all season (Table 2). Total *V*. *parahaemolyticus* levels also followed a seasonal trend. Generally, the levels of *V*. *parahaemolyticus* were at least 10-fold higher in the summer and autumn than in the cooler seasons in all four regions (Table 2). Both the prevalence and levels of *V*. *parahaemolyticus* were higher in the autumn than in the summer (Table 1).

**Table 2:** Regional and seasonal estimates of mean log *V*. *parahaemolyticus* densities in shellfish collected from different provinces of China.

Target / Season	Mean log *V*. *parahaemolyticus* densities (MPN g^-1^)^a^			
	Liaoning	Guangxi	Sichuan	Shandong
*tlh*				
Summer	0.49 A†	3.22 A‡	2.73 A‡	0.12 A†
Autumn	-	2.82 A†‡	2.68 A†	0.44 A§
Winter	0.16 A	-	1.38 B	-
Spring	-0.51†	1.44 B‡	1.61 B‡	-
*tdh*				
Summer	-0.05 A†	-0.71 A‡	-0.28 A†‡	-0.23 A†‡
Autumn	-	-0.68†	-0.58 AB†	-0.06 A†
Winter	-0.78 B†	-	-0.61 AB†	-
Spring	-0.58 AB†	-0.75 A†	-0.74 B†	-
*trh*				
Summer	0.10 B†	-0.77 A‡	-0.38 AB†§	-0.82 A‡§
Autumn	-	-0.81 A†	-0.50 AB†	-0.67 A†
Winter	-0.65 A	-	-0.02 A	-
Spring	-0.82 A†	-0.82 A†	-0.66 B†	-
ORF8				
Summer	-0.82 A†	-0.82 A†	-0.29 A‡§	-0.65 A†§
Autumn	-	-0.67 B†	-0.58 A†	-0.67 A†
Winter	-0.77 A	-	0.07 A	-
Spring	-0.74 A†‡	-0.81 A†	-0.41 A‡	-
Pathogenic				
Summer	0.12 B†	-0.71 AB‡§	-0.11 A†	-0.18 A†§
Autumn	-	-0.53 B†	-0.34 A†	-0.06 A†
Winter	-0.60 A	-	0.21 A	-
Spring	-0.58 A†	-0.75 A†	-0.41 A†	-

^a^ The same letter (A or B) within a column following the region/season estimate for each target indicates no significant differences (P > 0.05); the same symbol (†, ‡, or §) within a row following the region/season estimate for each target indicate no significant differences (P > 0.05).

**Fig 1:**
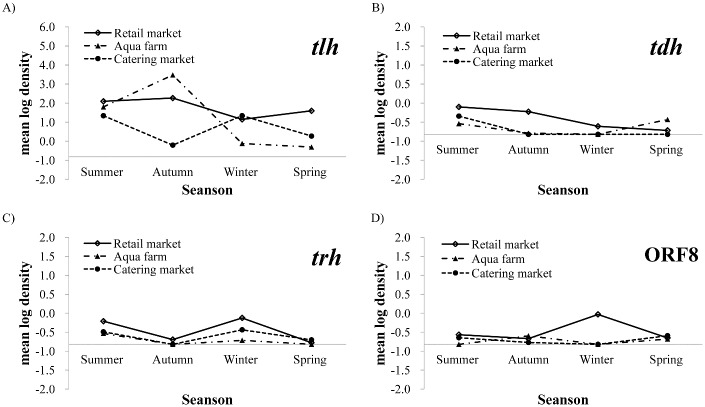
Seasonal trends in *V*. *parahaemolyticus* densities in shellfish collected from the three sections. The *X*-axis represents the level of *V*. *parahaemolyticus* in the four seasons. Different symbols indicate samples from retail markets, aqua farms, and catering markets. Total and pathogenic *V*. *parahaemolyticus* densities were determined by MPN-PCR for *tlh* (A), *tdh* (B), *trh* (C), and ORF8 (D). The *X*- and *Y*-axes intersect at reciprocal values of -0.82, which equals the log_10_ of 0.15 MPN g^-1^.

Fig 1A illustrates the mean total *V*. *parahaemolyticus* levels in shellfish collected from three market categories in different seasons. Overall, *V*. *parahaemolyticus* contamination was found in all three market categories in all seasons (Fig 1A). Aqua farms had a lower prevalence but higher abundance of total *V*. *parahaemolyticus* compared to retail markets (Table 1). Although catering markets had the lowest total *V*. *parahaemolyticus* densities, 20.0% of the samples exceeded 1,000 MPN g^-1^ (Table 1). Higher levels of *V*. *parahaemolyticus* in catering markets were found during the summer and winter, and the lowest values were found in autumn (Fig 1A). In the aqua farms and retail markets, the highest total *V*. *parahaemolyticus* levels were observed in the autumn than in the summer (Fig 1A).

The numbers of pathogenic *V*. *parahaemolyticus* ranged from < 0.3 to 2,400 MPN g^-1^; these numbers were generally lower than the total *V*. *parahaemolyticus* levels by several log units (Tables 1 and 2). The mean MPN level of pathogenic *V*. *parahaemolyticus* was 0.5 MPN g^-1^, with a standard deviation of 11.2 MPN g^-1^ (Table 1). In 58 (11.7%) samples, pathogenic *V*. *parahaemolyticus* densities were greater than 10 MPN g^-1^ (Table 1). Pathogenic *V*. *parahaemolyticus* levels showed no significant seasonal or regional trends. The highest levels of pathogenic *V*. *parahaemolyticus* were generally observed in the summer, following the prevalence trend (Table 2). However, a unique pattern was found in the samples from Sichuan, with the highest levels of *trh-* and ORF8-positive *V*. *parahaemolyticus* observed in the winter (Table 2). The geometric means of pathogenic *V*. *parahaemolyticus* in shellfish collected from the three market categories are presented in Fig 1B (*tdh*), C (*trh*), and D (ORF8). Pathogenic *V*. *parahaemolyticus* levels were generally below 1 MPN g^-1^ for all three markets and retail markets had more pathogenic *V*. *parahaemolyticus* than the other two market categories (Fig 1).

**Fig 2:**
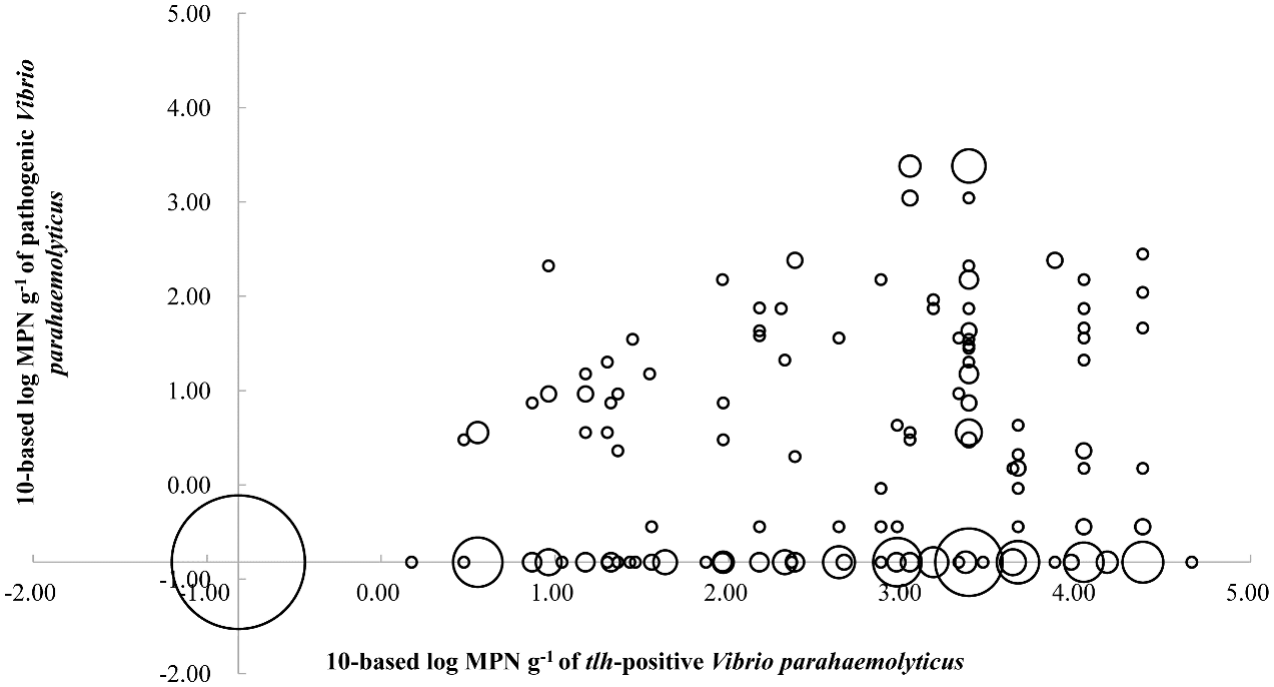
Relationship between the numbers of total and pathogenic *V*. *parahaemolyticus* in seafood. The *X*-axis represents the level of *tlh*-positive *V*. *parahaemolyticus*, whereas the *Y*-axis represents the level of pathogenic *V*. *parahaemolyticus*. The area of each circle represents the number of cases. The *X*- and *Y*-axes intersect at reciprocal values of -0.82, which equals the log_10_ of 0.15 MPN g^-1^.

Fig 2 shows the ratio of pathogenic to total *V*. *parahaemolyticus*, which varied widely. The correlation of log_10_-based MPN g^-1^ of pathogenic *V*. *parahaemolyticus* and total *V*. *parahaemolyticus* was significant (P = 0.01, Pearson’s correlation, two-tailed). The abundance of pathogenic *V*. *parahaemolyticus* tended to be higher in samples contaminated with high levels of total *V*. *parahaemolyticus*. The pathogenic genes were detected in 38.1% of the samples (72 out of 189) contaminated with levels of total *V*. *parahaemolyticus* greater than 1000 MPN g^-1^, whereas only 27.2% (40 out of 147) of samples contaminated with total *V*. *parahaemolyticus* levels less than 1000 MPN g^-1^ were pathogenic; these differences were revealed as significant by Pearson’s chi-squared test.

The multivariable models of the association between the occurrence of total and pathogenic *V*. *parahaemolyticus* with possible explanatory variables are shown in Table 3. Overall, there was a strong association between the occurrence of total *V*. *parahaemolyticus* and sampling site. The odds of samples from the Sichuan and Guangxi regions containing *V*. *parahaemolyticus* were 15.97- and 8.48-fold greater than those of samples from Shandong province, whereas the odds of samples from Liaoning province containing this pathogen were only 0.36-fold more likely than those of samples from Shandong province. No differences between clams and oysters were observed in the total *V*. *parahaemolyticus* model, although oysters showed 10-fold higher *V*. *parahaemolyticus* levels than did clams (93.3 MPN g^-1^ vs. 5.4 MPN g^-1^, respectively) (Table 1). A significant difference between the two species was observed for pathogenic *V*. *parahaemolyticus*, whereas no significant differences were observed among the sample sites. Significant differences among the market categories were observed for both the total and pathogenic *V*. *parahaemolyticus* models (P = 0.000 for both). For the total *V*. *parahaemolyticus* models, samples from aqua farms showed the highest odds, whereas for the pathogenic *V*. *parahaemolyticus* models, samples from retail markets gave the highest odds. A significant difference among different seasons was observed in the total *V*. *parahaemolyticus* model (P = 0.022), with samples obtained in the spring showing the lowest odds and those obtained in the summer showing the highest. Although not significant, similar trends were evident in the pathogenic *V*. *parahaemolyticus* model, with the exception that the highest odds appeared in the autumn.

**Table 3:** Multivariable models of association of the total and pathogenic *V*. *parahaemolyticus* levels with possible explanatory variables.

Variable(s)	All *V*. *parahaemolyticus*			Pathogenic *V*. *parahaemolyticus*		
	Odds ratio (95% CI)	Wald statistic P-value	LRS P-value	Odds ratio (95% CI)	Wald statistic P-value	LRS P-value
Sample sites						
Shandong	1.00		0.000	1.00		0.113
Liaoning	0.36 (0.14 0.93)	0.035		0.8 (0.32 1.99)	0.628	
Guangxi	8.48 (3.52 20.47)	0.000		0.45 (0.2 1)	0.049	
Sichuan	15.97 (6.2 41.09)	0.000		0.94 (0.43 2.05)	0.869	
Sample species						
Oysters	1.00		0.485	1.00		0.040
Clams	1.29 (0.63 2.65)	0.485		0.54 (0.3 0.98)	0.042	
Market categories						
Catering market	1.00		0.000	1.00		0.000
Retail market	8.72 (4.47 17.04)	0.000		2.88 (1.48 5.63)	0.002	
Aqua farm	12.85 (5.25 31.42)	0.000		0.79 (0.34 1.87)	0.598	
Seasons						
Winter	1.00		0.022	1.00		0.083
Spring	0.61 (0.26 1.43)	0.259		0.62 (0.27 1.42)	0.257	
Summer	1.71 (0.81 3.61)	0.163		1.18 (0.59 2.34)	0.643	
Autumn	1.51 (0.57 3.99)	0.410		1.76 (0.76 4.03)	0.184	

Environmental parameters were recorded in each of the two aqua farms of the Guangxi and Liaoning regions. Water temperatures ranged from 7.0 to 29.0°C and followed a typical seasonal progression. Water salinity in the Liaoning province ranged between 27 and 38 and remained relatively constant throughout the research period. Strong variation in water salinity was detected in the Guangxi province, ranging between 1 and 19. The log_10_ total *V*. *parahaemolyticus* levels of all 180 samples from the aqua farms were significantly associated (P < 0.01) with water temperature but not water salinity. The linear regression model that best fit the data was represented by the equation log_10_ (*V*. *parahaemolyticus* levels of the aqua farms) = 0.084 × water temperature (note that the constant parameter was not significant and was excluded from the equation). The model had an R^2^ value of 0.51, indicating that 51% of the observed variation in log_10_ total *V*. *parahaemolyticus* densities in shellfish was attributable to differences in water temperature. To investigate the effects of air temperature on total *V*. *parahaemolyticus* densities, a sharp rise in the mean levels of *V*. *parahaemolyticu*s was observed as the air temperature increased above 20°C, although substantial variability existed.

## Discussion

The aqua farms tested in this study were distributed over the three major sea areas of the Yellow Sea, Bohai Sea, and Beibu gulf, spanning from the north to the south. The retail markets and catering markets were distributed among all four provinces, including both coastal and inland areas. To the best of our knowledge, the data obtained from the three market categories in the four provinces represent the most comprehensive survey of *V*. *parahaemolyticus* abundance in shellfish in China to date. Moreover, the present study provides the first quantitative analysis of pathogenic *V*. *parahaemolyticus* in shellfish in China.

The prevalence of total *V*. *parahaemolyticus* in shellfish presented in this study (67.7%) was higher than values reported previously in studies of samples from China that were analyzed by direct counts using PCR or conventional culture procedures. The presence of *V*. *parahaemolyticus* in seafood in eastern China was determined to be 32.3% (97 out of 300) using the PCR assay directly without overnight MPN enrichment, compared to 26.0% (78 out of 300) using conventional culture [24]. Yang et al. reported that approximately 33.4% (164 out of 491) of fresh seafood samples were contaminated with *V*. *parahaemolyticus* between July and October 2007 in Jiangsu province and Shanghai city in China using a conventional culture procedure [25].

The multiplexed real-time PCR method used in the present study is capable of detecting an initial inoculum of 1 CFU of *V*. *parahaemolyticus* per gram of oyster tissue homogenate after overnight enrichment [23]. Similar studies have shown that the sensitivity of MPN-PCR is 100-fold higher than that of direct count measurements using PCR or the conventional MPN culture procedure [24, 26–28]. This sensitivity difference may partly explain why the densities of *V*. *parahaemolyticus* reported here are much higher than those previously reported based on direct PCR or the culture-based method. Our results are similar to those of a report analyzing seafood from Qingdao city in China, in which MPN-PCR was used to examine 225 samples in different seasons. The prevalence of total and *tdh-*positive *V*. *parahaemolyticus* was determined to be 73.3% and 41.5%, respectively [28]. In addition to variability in the methodology, differences in *V*. *parahaemolyticus* levels among studies may reflect differences in the spatial and temporal characteristics of sampling. In the present study, the Guangxi and Sichuan provinces of southern China presented 100-fold higher *V*. *parahaemolyticus* levels than the northern provinces, including Liaoning and Shandong. In the summer and autumn, when the temperatures were higher, shellfish bore higher *V*. *parahaemolyticus* levels than in the spring and winter. These spatial and temporal characteristics may be attributed to one factor (temperature), as southern China has a higher average air temperature than the northern areas. Differences in density among studies can also result from differences in post-harvest multiplication.

The prevalence of pathogenic *V*. *parahaemolyticus* samples in our study was in agreement with the results of some recent studies in other countries [29–31], although the species investigated as well as the sampling locations differed. However, few studies are available concerning the levels of pathogenic *V*. *parahaemolyticus* in shellfish around the world. DePaola et al. found that the average number of *tdh*-positive *V*. *parahaemolyticus* in oysters collected from Alabama was approximately 2 CFU g^-1^ [8]. The level of *tdh-*positive *V*. *parahaemolyticus* in oysters in Chesapeake Bay was found to be 10 CFU g^-1^ [32]. Although a slightly lower average number of pathogenic *V*. *parahaemolyticus* (0.5 MPN g^-1^) was found in shellfish in this study than in these US studies, 11.6% of samples exceeded 10 MPN g^-1^. Generally, the levels of *V*. *parahaemolyticus* in shellfish marketed for consumption may be used to estimate the potential risk of gastroenteritis. However, the number of virulent *V*. *parahaemolyticus* is a better indicator of public health risk [33]. Attention is strongly required when the level of total *V*. *parahaemolyticus* exceeds the limit of 10^4^ bacteria per gram and/or the level of pathogenic *V*. *parahaemolyticus* exceeds 10 bacteria per gram in environmental shellfish [2, 34]. Our results suggest that contamination assessment and risk prediction of shellfish using total *V*. *parahaemolyticus* counts is insufficient and that pathogenic levels should receive more consideration. The positive correlation of pathogenic and total *V*. *parahaemolyticus* abundance in our study may shed light on this analysis. There were no marked seasonal or regional trends concerning pathogenic *V*. *parahaemolyticus* levels, although trends may be obscured by low detection frequency and densities.

The levels of *V*. *parahaemolyticus* in shellfish could change dramatically depending on the handling practices, and cross contamination could occur at any phase of the long processing and distribution chain [35]. The most appropriate evaluation of the performance of the overall control of the pathogen should be performed at the point of consumption. However, data obtained from other market categories help to identify how each part of the control system affects bacterial growth. Our data showed that the aqua farms had a lower prevalence but higher abundance of *V*. *parahaemolyticus* than the retail markets. These findings suggest that the aqua farms may have served as the major source of *V*. *parahaemolyticus* contamination, whereas the retail markets were more responsible for the spread of the bacteria. However, these findings do not rule out the possibility of a sporadic distribution of *V*. *parahaemolyticus* in nature. Evidence for a sporadic distribution is seen in the multivariable total *V*. *parahaemolyticus* models, which showed that the samples collected from the aqua farms presented the highest odds of contamination. This finding contradicts the conclusion drawn by the Food and Drug Administration (FDA) in 2005, who proposed that the highest risk of *V*. *parahaemolyticus* illness is attributed to post-harvest growth in oysters [35]. Although the samples from the catering markets had the lowest levels of bacteria, possibly due to cooking, 20.0% of the samples exceeded 1,000 MPN g^-1^ and the Chinese food safety standard GB 29221 for *V*. *parahaemolyticus* limits in ready-to-eat seafood [36]. Therefore, the Chinese standard concerning the microbiological quality of ready-to-eat seafood does not provide sufficient protection and requires revision to include monitoring of both total and pathogenic *V*. *parahaemolyticus* in harvesting areas and retail markets.

The multivariable model of the association between the occurrence of pathogenic *V*. *parahaemolyticus* and possible explanatory variables showed that samples collected from retail markets presented the highest odds of contamination. This result may be due to the decrease in total *V*. *parahaemolyticus* numbers or the differences in the survival and tolerance of virulent and avirulent strains during the processing and distribution chain.

Iced seafood is typically considered to have little chance of transmitting *V*. *parahaemolyticus* because this bacterium is sensitive to low temperatures and can be progressively inactivated [37, 38]. However, we found that 87.8% of iced shellfish sampled in Sichuan province were contaminated with *V*. *parahaemolyticus*; therefore, the risk of *V*. *parahaemolyticus* disease could increase when the food is mishandled or preserved under conditions that benefit the growth of bacteria. *V*. *parahaemolyticus* was found to be the most prevalent cause of outbreaks of bacterial foodborne disease in littoral zones but only the third most prevalent cause in inland provinces in China [39]. Although directly correlating foodborne disease data with the food contamination data of *V*. *parahaemolyticus* in shellfish is difficult, our study may bring both the littoral and inland areas into focus.

Pathogenic *V*. *parahaemolyticus* can be detected regardless of season and region. The highest levels of *trh-* and ORF8-positive *V*. *parahaemolyticus* were found in the winter in other similar study [40]; this result may be related to the uneven contribution of pathogenic samples throughout the year. Therefore, the winter cannot be considered an absolutely safe season. Oysters showed higher levels of total and pathogenic *V*. *parahaemolyticus* than clams, and therefore, more attention should be paid to oysters.

We found that the abundance of *V*. *parahaemolyticus* in the aqua farms was affected by water temperature but not by water salinity, which was partially consistent with previous studies [8, 41]. These findings indicate that temperature is a major factor in both the seasonal and regional distribution of *V*. *parahaemolyticus* in shellfish-growing areas.

## Conclusions

This study is the first to simultaneously examine the prevalence and abundance of total and pathogenic *V*. *parahaemolyticus* in shellfish from three sections of the food supply chain in four provinces of China. Relative to previous reports, significantly elevated levels of total and pathogenic *V*. *parahaemolyticus* were found in our study. Exceeding the limit for *V*. *parahaemolyticus* set by the Chinese food safety standard GB 29221 for ready-to-eat seafood exposes Chinese consumers to a high potential risk of *V*. *parahaemolyticus*-related gastroenteritis. Emphasis should be placed on oysters for risk control of *V*. *parahaemolyticus*, particularly during the warmer months of the year, in retail markets and in southern China, including the inland cities.

In summary, the results of this study highlight the presence of both total and pathogenic *V*. *parahaemolyticus* in three sections of the food supply chain in four provinces of China over all four seasons, indicating a potential public health hazard. Chinese-specific risk assessment of *V*. *parahaemolyticus* in shellfish requires quantitative data on the organism, particularly on the pathogenic strains. Thus, surveillance of both total and pathogenic *V*. *parahaemolyticus* in shellfish is crucial to obtain additional reliable data that can be used to conduct risk assessments and to evaluate the efficiency of controls aimed at reducing exposure to and risk of *V*. *parahaemolyticus*.

## Supporting Information

S1 File This file contains additional sampling information and other supplementary data. (XLS)
